# Environmental DNA reflects spatial and temporal jellyfish distribution

**DOI:** 10.1371/journal.pone.0173073

**Published:** 2017-02-28

**Authors:** Toshifumi Minamoto, Miho Fukuda, Koki R. Katsuhara, Ayaka Fujiwara, Shunsuke Hidaka, Satoshi Yamamoto, Kohji Takahashi, Reiji Masuda

**Affiliations:** 1 Graduate School of Human Development and Environment/Faculty of Human Development, Kobe University, Kobe City, Hyogo, Japan; 2 Maizuru Fisheries Research Station, Kyoto University, Maizuru City, Kyoto, Japan; University of Hyogo, JAPAN

## Abstract

Recent development of environmental DNA (eDNA) analysis allows us to survey underwater macro-organisms easily and cost effectively; however, there have been no reports on eDNA detection or quantification for jellyfish. Here we present the first report on an eDNA analysis of marine jellyfish using Japanese sea nettle (*Chrysaora pacifica*) as a model species by combining a tank experiment with spatial and temporal distribution surveys. We performed a tank experiment monitoring eDNA concentrations over a range of time intervals after the introduction of jellyfish, and quantified the eDNA concentrations by quantitative real-time PCR. The eDNA concentrations peaked twice, at 1 and 8 h after the beginning of the experiment, and became stable within 48 h. The estimated release rates of the eDNA in jellyfish were higher than the rates previously reported in fishes. A spatial survey was conducted in June 2014 in Maizuru Bay, Kyoto, in which eDNA was collected from surface water and sea floor water samples at 47 sites while jellyfish near surface water were counted on board by eye. The distribution of eDNA in the bay corresponded with the distribution of jellyfish inferred by visual observation, and the eDNA concentration in the bay was ~13 times higher on the sea floor than on the surface. The temporal survey was conducted from March to November 2014, in which jellyfish were counted by eye every morning while eDNA was collected from surface and sea floor water at three sampling points along a pier once a month. The temporal fluctuation pattern of the eDNA concentrations and the numbers of observed individuals were well correlated. We conclude that an eDNA approach is applicable for jellyfish species in the ocean.

## Introduction

Environmental DNA (eDNA) represents all types of DNA present in the environment, DNA from organisms themselves and extracellular DNA. eDNA approaches for mapping the distribution of organisms were first used for microorganisms [1], and recently, they have been applied to macro-organisms [2–4]. It is estimated that macro-organisms release their DNA via secretions and excretions, and that the main form of the eDNA would be derived from organelles [5]. The presence or absence of the eDNA for a specific organism generally indicates the presence or absence of that organism, and the eDNA concentration is considered to reflect the biomass or abundance of the target organisms [6–8]. In terms of cost, labor, and sensitivity, this relatively new method for determining species distribution is reported to outperform the traditional survey methods including direct catch by angling and netting, direct observation by diving, and indirect observation by filming and echo sounding [9]. To date, most eDNA surveys were conducted in freshwater mesocosms [10,11], streams and rivers [6,12–14], ponds and lakes [15–17], and a few eDNA surveys in oceans have been reported [8,11,18–20].

Explosive increases of jellyfish, here defined as cnidarian medusa, have been reported worldwide in recent years [21]. Various anthropogenic effects including climate change, eutrophication, architectural structures, and overfishing are causes of such explosive population increases [22], which sometimes bring damage to fisheries, injuries to humans, and cooling-water trouble for power plants [23]. To reduce these negative effects of jellyfish outbreaks, the distribution and seasonality of harmful species should be clarified. However, such basic information for most jellyfish species is unknown.

In the present study, we developed eDNA quantification methods for jellyfish species using Japanese sea nettle (*Chrysaora pacifica*) as a model species. *Chrysaora pacifica* belongs to Pelagiidae (Semaeostomeae) and possesses an umbrella of up to 20 cm in diameter and tentacles as long as 72 cm [24]. On the coast of Japan, the number of *C*. *pacifica* jellyfish has increased in recent years [25], and there have been reported cases of severe injuries inflicted on swimmers by *C*. *pacifica* [26]. If we know the spatial and temporal distribution patterns of this harmful jellyfish species more precisely, we would be able to mitigate the damage by local expelling or by selectively avoiding the outbreak season or location. Thus, eDNA detection and/or quantification of *C*. *pacifica* could provide valuable information for future control of jellyfish outbreaks.

To this end, we first established an eDNA quantification method for *C*. *pacifica* with a TaqMan real-time PCR system in a tank experiment. Next, we performed spatial and temporal distribution surveys for this species in the Maizuru Bay where *C*. *pacifica* is abundant, and the results were compared to the distribution patterns inferred from direct visual observation. Using these adapted protocols, we successfully detected jellyfish eDNA in both surface and sea floor water samples.

## Materials and methods

### 2.1 Ethics statement

Field research in the present study was approved by the harbormaster of Maizuru Bay (Permission Number 191 issued on June 9, 2014).

### 2.2 Designing species-specific primers and probe

To design primers and a probe specific to *C*. *pacifica*, we sequenced the partial COI region of the species (11 individuals) and *Aurelia* sp. (three individuals), which is the closest related jellyfish to our target species in the surveyed area. Jellyfish were caught from a pier of Maizuru Fisheries Research Station (MFRS), Kyoto University (35°29′N, 135°22′E) and the total DNA was extracted from tissue samples using DNeasy Blood and Tissue Kit (Qiagen, Hilden, Germany) according to the manufacturer’s protocol. Because there was no available sequence in public databases for these two jellyfish species collected from the surveyed area, we first amplified and sequenced the partial COI regions with the primers Jellyfish_CO1_F 5′-KKTCAACAAAYCATAAAGATATWGG-3′ and Jellyfish_CO1_R2 5′-GGAACTGCTATWATCATWGTWGC-3′, both of which were modified from previously reported primers (Folmer et al., 1994). Based on the consensus sequence for *C*. *pacifica*, candidate primers and probes were identified using Primer Express 3.0 software with default parameters (Thermo Fisher Scientific, Waltham, MA). After comparing the COI sequences of *C*. *pacifica* and *Aurelia* sp., the primer sets containing a total of five species-specific variations were selected for both primers, within five bases from the 3ʹ ends. The sequences of the designed specific primers and probe are as follows: Cpa_COI_F primer (5′-CCCAGATATGGCTTTTCCTAGA-3′), Cpa_COI_R primer (5′-TGAGTGAGCTTGTATAGCTGATA-3′), and Cpa_COI_P probe (5′-FAM-TAGGATCCTCCCTAATTG-NFQ-MGB-3′). To check the specificity of the primers and probe, we performed real-time PCR by using the total DNA of *Aurelia* sp. The real-time PCR conditions are same as those described in section 2.7.

### 2.3 Tank experiment

The tank experiment was performed at MFRS. Three 500 L tanks were used, and each tank was filled with 500 L of seawater, which was pumped from an area adjacent to MFRS and was filtered using a sand filter system (5G-ST, Nikkiso Eiko, Japan). Seawater was continuously poured into the tank (1.4 L min^-1^), and was discharged from the overflow drainage. The water temperature was not controlled and showed slight fluctuation during the experimental period (17.2–20.4°C, 17.1–20.4°C, and 17.2–20. 4°C in tanks 1, 2, and 3, respectively); no aeration was performed. To avoid affecting eDNA release rate, animals were starved during the experiment. Jellyfish individuals were caught at the pier of MFRS by hand net, and by using buckets, they were gently transferred to the tanks. Afterward, three individuals were placed into each experimental tank at 16:00, which was set as Time 0. The umbrella diameters of the nine individuals at the start of the experiment were 13.4 ± 1.51 (mean ± 1 standard deviation [SD]) cm. At 0 (just after introduction of individuals), 1, 2, 4, 8, 16, 24, 48, 72, 120, and 168 h after introduction of jellyfish, 1 L of drainage water was collected and immediately filtrated with glass fiber filters (0.7 μm mesh; GE Healthcare Japan, Tokyo). To avoid contamination between samples, all equipment for water collection and filtration was bleached in 0.1% sodium hypochlorite before use. Because the water used to fill the tanks was obtained from Maizuru Bay, where the target species is abundant, the filtered seawater could have contained remnant DNA from this species. Therefore, just before and after the experiment, 1 L of the pouring water was collected and eDNA was measured to obtain a background value. An extraction blank, i.e. 1 L of autoclaved artificial seawater (New Marin Merit, Matsuda, Japan) prepared with distilled water was filtered in the same manner after filtration of the samples at each sampling interval to monitor cross-contamination between samples. The glass filters were immediately stored at –25°C until further analysis. The umbrella diameter (10.9 ± 0.86 cm) and wet weight (94.6 ± 26.0 g) of each individual were measured after the tank experiment.

To evaluate the degradation rate of the eDNA, 3 L water from each tank was collected at 72 h after the start of the experiment, stored in sealed plastic bags, and incubated in another tank. The temperature was not controlled (19.5–20.4°C) and no aeration was performed. At 48 and 96 h after the collection of this water, 1 L of each incubated water sample was filtered as described in the previous paragraph.

### 2.4 A bay-scale spatial survey in Maizuru Bay

We conducted a field survey of jellyfish in Maizuru Bay on June 18, 2014, where a survey for Japanese jack mackerel (*Trachurus japonicus*) was concurrently conducted [8]. At each corner of the 400 m mesh grids, 47 water sampling sites were set throughout the bay (see Figure 1 in Yamamoto et al. 2016). The water sampling started at 09:48 and ended 15:57. The water temperatures at the surface and bottom were 23.9 ± 0.30 and 21.7 ± 0.54 (mean ± 1 SD, *N* = 47), respectively. The eDNA samples used in this study were the same as those used in Yamamoto et al. (2016). Briefly, seawater samples were collected both from the sea surface using a bucket and from ~1.5 m above the sea floor using a van Dorn water sampler at each site in Maizuru Bay. Because jellyfish are usually present not only at the water surface but also at various depth in the water column, we collected water from the surface and the sea floor. Each 1 L water sample was immediately filtered on board through a glass fiber filter of 0.7 μm average pore size. To minimize cross-contamination, the bucket was washed twice or more with surface water at each sampling site and the van Dorn sampler was placed in sea floor water for several minutes before each water collection. The filter funnels and measuring cups used for filtration were bleached after every filtration. In addition, to verify the effectiveness of the decontamination procedure, we filtered artificial seawater with a randomly selected filter funnel and measuring cup at every fifth site to serve as a negative equipment control. We collected eDNA samples in triplicate at each site, where one sample was used for eDNA quantification.

The numbers of *C*. *pacifica* individuals within 5 m from the end of the starboard side and within 1 m depth from the surface were counted by an observer while the research vessel moved between eDNA sampling stations. The observer was trained to count jellyfish in a preliminary survey cruise with the help of a tape measure to evaluate the distance from the research vessel.

For geographic information system (GIS) analysis, the accumulated number of *C*. *pacifica* individuals counted between two water sampling points was treated as the number of individuals at the center of two points.

### 2.5 Temporal survey at a pier in Maizuru Bay

Temporal surveys combining direct observation and eDNA quantification were conducted from March to November 2014. Nine water samplings were performed on March 4, April 2, May 14, June 3, July 2, August 14, September 10, October 24, and November 20 at the pier of the MFRS. Six 1 L water samples, three from the surface water and three from the sea floor, were collected in each survey. The methods used for water collection and filtration were the same as described for the spatial survey in section 2.4. As an extraction blank, 1 L autoclaved artificial seawater was filtered on each sampling day.

The numbers of jellyfish including *C*. *pacifica* have been monitored by one of the authors (R.M.) virtually every morning at 8:00 AM from the pier of MFRS since June 2002. The data from March to November 2014 of this ongoing data collection were used for comparison with eDNA concentration data. Over these 275 days, data were missing for 5 days. In each observation, jellyfish were counted within a distance of 3 m from the pier, thus encompassing an area of approximately 150 m^2^, and 1 m in depth.

### 2.6 DNA extraction from the filters

DNA trapped on the filters obtained from the tank experiments, bay-scale spatial survey, and temporal survey were extracted using DNeasy Blood and Tissue Kit using a protocol modified from that previously reported [8]. Briefly, a sample filter was placed into a Salivette tube (Sarstedt, Nümbrecht, Germany). After 20 μL Proteinase K and 400 μL buffer AL (Qiagen) were added, the tube was incubated for 30 min at 56°C. The liquid held in the filter was collected by centrifuge. Then, 200 μL of tris-ethylenediaminetetraacetic (TE) buffer was put on the filter, and the liquid was again collected by centrifuge. Buffer AL (200 μL) and absolute ethanol (600 μL) were added to the collected liquid and the mixture was transferred to a DNeasy spin column. We followed the manufacturer’s instructions for subsequent steps, and finally, eDNA was eluted in 100 μL AE buffer instead of the manufacturer’s recommended 200 μL to obtain a more concentrated eDNA solution.

### 2.7 Quantification of *C*. *pacifica* eDNA

The eDNA concentration was quantified by qPCR using a StepOnePlus Real-Time PCR System (Thermo Fisher Scientific, Waltham, MA). Each reaction contained 900 nM primer and 125 nM TaqMan Probe in 1× TaqMan gene expression master mix (Thermo Fisher Scientific) and 2 μL of DNA sample to reach a final volume of 13.3 μL. Cpa_COI_F and Cpa_COI_R primers and a Cpa_COI_P probe were used for the quantification of *C*. *pacifica* mitochondrial DNA. The sequences of the primers and probe are presented in the results. Dilution series containing 2 × 10^1^ to 2 × 10^4^ copies per PCR tube were prepared and used as quantification standards. DNA of partial COI (231 bp) were cloned into pUC57 plasmids and amplified in triplicate to obtain a standard curve. The qPCR conditions were as follows: 2 min at 50°C, 10 min at 95°C, 40 cycles of 15 s at 95°C, and 60 s at 60°C. Three replicates were used for each sample, and three replicate negative controls containing ultrapure water instead of template DNA were included in all PCR plates. Previously described precautions were adopted to avoid carry-over contamination [12].

### 2.8 Data analysis

For the tank experiment, the amount of eDNA released by each jellyfish within a given time interval (*t*) was calculated as described by Ellis et al. (2004)[27], whose method was originally developed for evaluating the release rate of cortisol, as follows: *H*_*t*_ = *Vkt* (*C*_*t*_—*C*_*0*_e^-*kt*^)(1—e^-*kt*^)^-1^, where *H*_*t*_ (DNA copies) is the released copy number of DNA within time interval *t*, *V* is the water volume, *C*_*0*_ and *C*_*t*_ represent the DNA concentrations at the start and end of the two sampling periods (over time interval *t*), and *k* is the instantaneous rate of decrease due to dilution from the inflow water. Values for *k* (min^-1^) were derived as *RV*^-1^, where *R* is the water inflow rate. The averaged water residence time in the tank was 6.0 h.

For the spatial survey, we examined the relationship between eDNA concentration and observed jellyfish abundance using a generalized linear model (GLM) with gamma error (inverse link). In the model, logarithms of eDNA copy numbers in each PCR at the surface and sea floor water were explanatory variables, and estimated abundance of the jellyfish at sampling points was the response variable. Abundance at sampling points was estimated as an extracted value using spline interpolation based on the observed number of jellyfish individuals, which was conducted by the open source software GLASS in the geometric information system QGIS ver. 2.10.1. If the estimated value was negative, we changed it into a negligible positive value (10^−6^) to enable analysis using gamma distribution. For each explanatory variable, we examined the significance and multicollinearity using a Wald test and variance inflation factor (VIF), respectively. The eDNA concentration in surface and sea floor water samples were compared by Wilcoxon signed rank test by using the ‘exactRankTests’ function (http://cran.ism.ac.jp/) in R.

For the temporal survey, the Spearman’s rank correlations among the sequential data of *C*. *pacifica* eDNA at six environmental points were investigated. We performed all analyses using the statistical software package R ver. 3.1.1 [28].

## Results

### 3.1 Designing the primers and probe for Japanese sea nettle

The partial COI regions (ca. 800 bp) of *C*. *pacifica* caught in Maizuru Bay were amplified and sequenced. The sequences were deposited to the DNA database of Japan (DDBJ), and the accession numbers are LC191567–77.

To check the specificity of the primers and probe, we performed real-time PCR using total DNA of *Aurelia* sp. as templates, and no amplification signal was obtained. In addition, we sequenced the 16 and 10 amplicons of the spatial distribution survey and temporal distribution survey, respectively, and confirmed that these amplicons were all derived from the target species.

### 3.2 Quantification of eDNA copy numbers

In all the runs, R^2^ values of calibration curves were more than 0.994, the range of slopes, Y-intercept, and PCR efficiency were between –3.583 and –3.386, 40.145 and 41.161, and 0.901 and 0.974, respectively. Based on the calibration curve of each run and the Ct value of each sample, the copy number of gene fragment of COI of *C*. *pacifica* in each PCR reaction (copies) and the eDNA concentration in the sample water (copies L^-1^) were calculated.

### 3.3 Tank experiments

The umbrella diameters of the individuals at the end of the experiment were 10.9 ± 0.86 (mean ± 1 SD) cm, showing ca. 20% decrease over the experiment representing the starvation occurring during this period.

**Fig 1:**
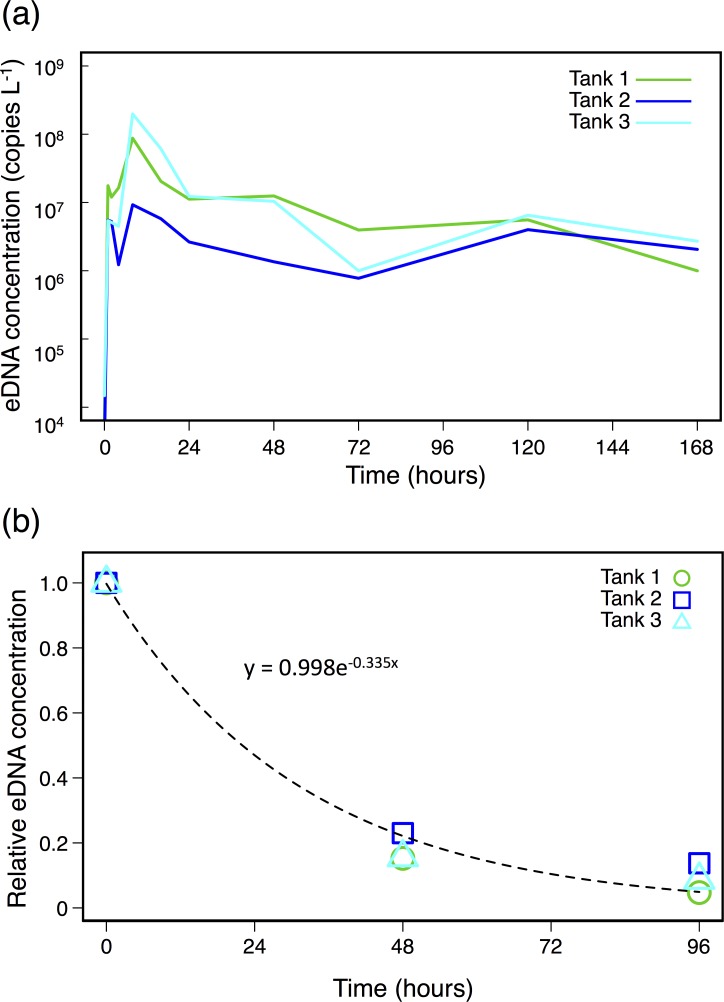
The results of the tank experiments. (a) Time dependent change of eDNA concentration (copies L^-1^) in the tank experiment. (b) Relative eDNA concentration, i.e., the concentration at time *t* divided by the initial concentration in the tank, represents the combined exponential degradation in all three tanks. The exponential regression curve for the eDNA degradation was y = 0.998e^-0.0335x^ (where x and y mean time in hour and relative DNA concentration at time x, respectively).

The eDNA of Japanese sea nettle was detected from all three tanks at every time point. The eDNA concentration varied at 5.47 × 10^3^–8.74 × 10^7^, 2.40 × 10^3^–9.26 × 10^6^, and 1.48 × 10^4^–1.99 × 10^8^ copies L^-1^ in tanks 1, 2, and 3, respectively (Fig 1A). The eDNA concentration peaked twice, at 1 and 8 h after the start of the experiment although the change was not statistically significant. Three of ten extraction controls showed a positive signal, but the signal was low and was under the quantification limit (50 copies per PCR tube). A small amount of eDNA might be contaminated during DNA extraction process, because large amounts of eDNA were collected in tank experiments. We detected quantifiable concentrations of eDNA from the tank inlet water (7.5 × 10^3^ ± 2.1 × 10^3^ copies L^-1^ [mean ± 1 SD], *N* = 6), and the background level did not vary among three tanks and among time points. The concentration of the jellyfish eDNA in the inlet water was less than 1% of that of corresponding tank water samples. Therefore, we conclude that the background DNA largely does not affect the results of tank experiments. The calculated eDNA release rates varied highly among tanks.

Jellyfish eDNA showed ~90% degradation in four days. The exponential regression curve for the eDNA degradation was y = 0.998e^-0.0335x^ (where x and y represent time in hour and relative DNA concentration at time x, respectively). From this regression, the degradation rate was 0.0329 (± 0.0027 [1 standard error]) h^-1^ (Fig 1B).

### 3.4 Spatial survey in Maizuru Bay

**Fig 2:**
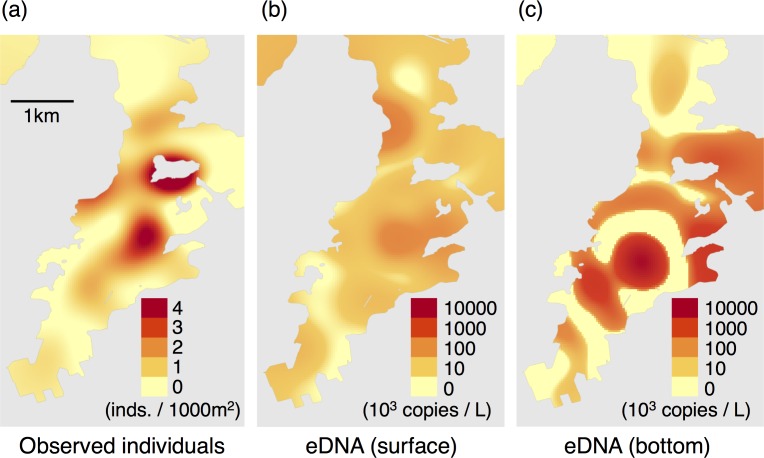
The results of the spatial survey. (a) The estimated abundance of *C*. *pacifica* at the day of survey based on the visual survey. (b) The concentration of *C*. *pacifica* eDNA at the surface and (c) on the sea floor.

**Fig 3:**
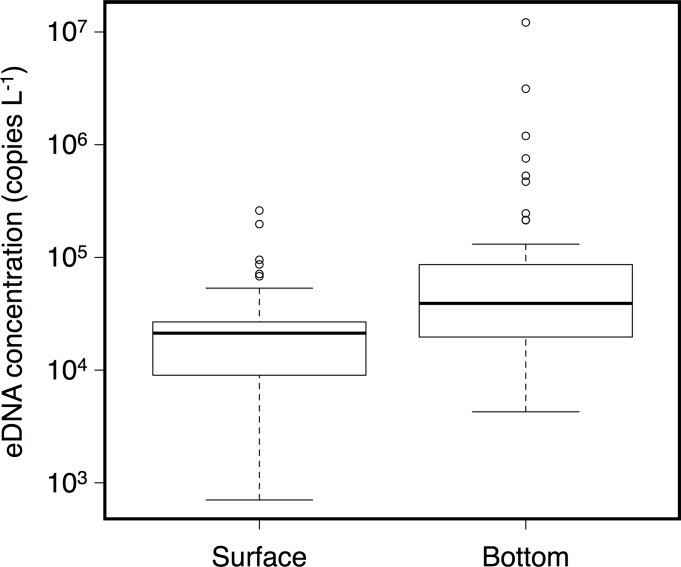
The comparison between eDNA concentrations of surface and sea floor samples. The eDNA concentrations in the sea floor were significantly higher than that of surface samples (Wilcoxon signed rank test, *P* = 0.00051).

The eDNA of *C*. *pacifica* was detected in all 94 samples (two depths × 47 points). The eDNA concentration was 7.05 × 10^2^–2.60 × 10^5^ copies L^-1^ and 4.26 × 10^3^–1.21 × 10^7^ copies L^-1^ for the surface and sea floor water samples, respectively (Fig 2). The copy numbers of *C*. *pacifica* eDNA were ~13 times higher in sea floor water samples than in surface water (Fig 3). The eDNA concentrations were significantly higher in the sea floor samples than in the surface samples (Wilcoxon signed rank test, *P* = 0.00051). According to the GLM analysis, estimated abundance based on count data showed a positive correlation with eDNA copy number in surface water (estimated coefficient = -0.171, *P* = 0.007) while it showed no significant correlation with eDNA copy number in sea floor water (*P* = 0.30). No multicollinearity was suggested between surface and sea floor water eDNA copy numbers (VIF = 1.14).

### 3.5 Temporal survey at the pier

**Fig 4:**
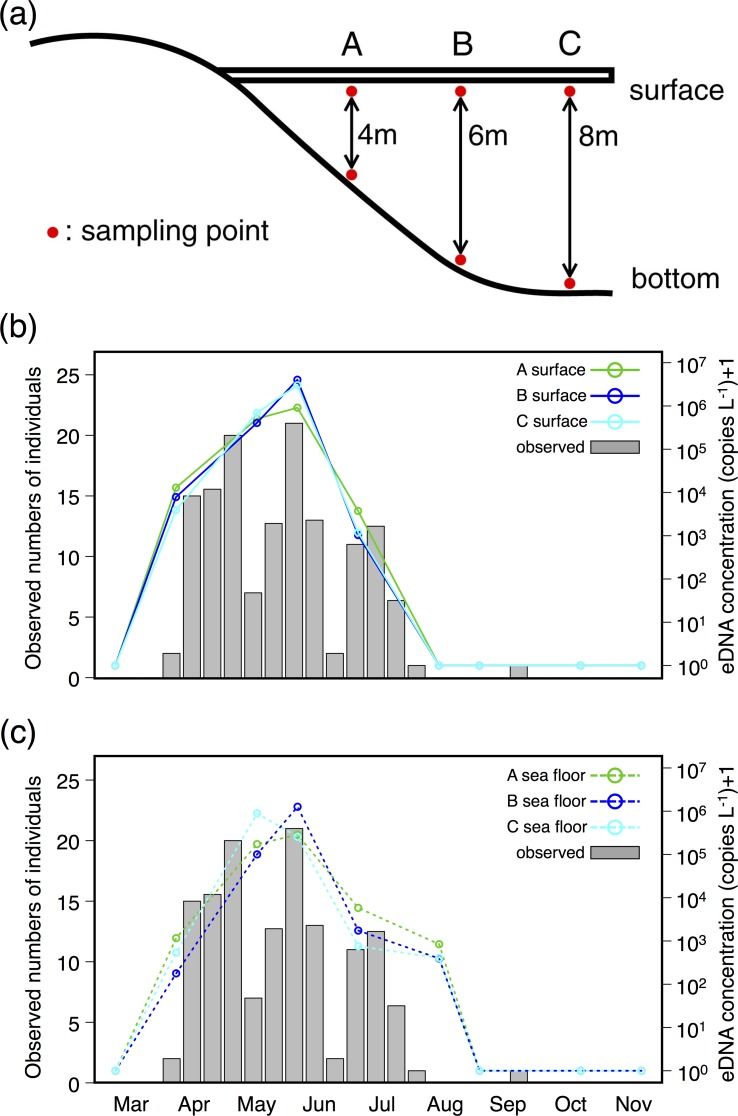
The schematic drawing of the sampling point for temporal survey and the results. (a) The schematic drawing of the sampling point for temporal survey conducted at the pier of Maizuru Fisheries Station, Kyoto University. The surface and sea floor water samples were collected at three (A, B, and C) points where the water depth is 4, 6, and 8 m, respectively. (b) The temporal change of observed numbers of *C*. *pacifica* in the visual survey and the eDNA concentration in each surface sample. (c) Temporal change in observed numbers of *C*. *pacifica* in the visual survey and eDNA concentration in each sea floor sample. Note that the observed numbers of individuals were presented as the average of 10 days and that the same observation data were presented in (b) and (c) for better visibility.

The eDNA copy numbers of *C*. *pacifica* in specimens collected from the pier varied from under the quantification limit to 4.03 × 10^6^ copies L^-1^ (Fig 4). All 15 combinations among six datasets showed significant correlation (Table 1). The correlations between averaged eDNA concentrations of the six samples and observed numbers of individuals on the sampling day were not significant (*R* = 0.157, *P* = 0.686); however, the averaged eDNA concentrations of the six samples showed significant correlation when the observed numbers of individuals were averaged on the sampling day and the previous several days. The highest correlation was detected when the observed numbers of individuals were averaged for five days (the sampling day and the previous four days) (*R* = 0.934, 0.934, 0.948, 0.965, and 0.843, *P* = 0.00023, 0.00023, 0.000098, 0.000025, and 0.0043 for averaging observed numbers of individuals on the sampling day and the previous 1, 2, 3, 4, and 5 days, respectively).

**Table 1:** The correlation coefficients among six eDNA sequential samples of the temporal survey.

	A-sea floor	A-surface	B-sea floor	B-surface	C-sea floor	C-surface
**A-sea floor**	—					
**A-surface**	0.934**	—				
**B-sea floor**	0.982**	0.858**	—			
**B-surface**	0.934**	1.00**	0.858**	—		
**C-sea floor**	0.982**	0.915**	0.964**	0.915**	—	
**C-surface**	0.934**	1.00**	0.858**	1.00**	0.915**	—

** *P* < 0.01

## Discussion

We succeeded in detecting and quantifying eDNA of *C*. *Pacifica*, a jellyfish species. To the best of our knowledge, this is the first report of eDNA detection in jellyfish. The eDNA detection of broad taxa including mammals, amphibians, fish, shellfish, arthropods, flatworms, and plants have been reported to date [14,18,19,29–32], and the addition of jellyfish to these eDNA-applicable taxa suggests that eDNA approaches are useful for a wide range of macro-organisms. The availability of an eDNA approach will provide new options for surveying jellyfish, and may contribute to mitigation of the damages caused by harmful jellyfish.

The results of the tank experiment revealed that the eDNA release rate of jellyfish was higher than those reported for fishes. Compared to the third and fourth day results of bluegill sunfish (*Lepomis macrochirus*) kept in tanks [33], the averaged eDNA release rate of a *C*. *pacifica* individual (94.6 ± 26.0 g in wet weight) from the second to seventh day of the experiment, when the eDNA release rate seems stabilized (~1.53 × 10^8^ copies h^-1^ per individual), is 8.8 and 105 times greater than that of adults (30–75 g in wet weight) and juveniles (0.5–2.0 g), respectively. Similarly, the eDNA release rate of *C*. *pacifica* is 150–253 and 52–206 times greater than those of the sub-adult bighead carp (200–1600 g) and silver carp (100–1000 g), respectively (the mass of the individuals were read from Figure 2 of Klymus et al. [34]). Note that we did not compare the eDNA release rate per weight because of the high water content in jellyfish (ca. 96% in *C*. *pacifica*, [35]). Although eDNA release rate may differ by species, size, and physiological and behavioral characteristics, the prospect of eDNA detection of jellyfish is promising compared to fish species because of its higher eDNA release rate.

The concentration of eDNA varied considerably among tanks, showing as much difference as two orders of magnitude (Fig 1A). Neither a relationship between eDNA concentration and body size nor a change in size during experiments was observed, and the background eDNA concentration in the inlet water did not vary among tanks. Therefore, the eDNA release rate might be highly variable among individuals.

In all three tanks, the eDNA release rate peaked at 1 and 8 h after the start of the experiment, and then decreased and stabilized at 48 h or later. The first peak at 1 h after the start of experiment may be caused by handling stress as discussed in other species [4,11,33,34], whereas we have no explanation for the second peak observed at 8 h after the start. The eDNA concentration in the inlet water might have varied among time points, because we checked the concentration only at Time 0 and Day 7. However, considering its relatively low value and variability, it is unlikely that eDNA from the inlet water could have introduced a systematic bias on the present result.

The degradation rate of eDNA of *C*. *pacifica* was 0.0329 (per hour). The rate is comparable to that of marine fishes (0.0133 for European flounder *Platichthys flesus* and 0.0288 for three-spined stickleback *Gasterosteus aculeatus* calculated from Thomsen et al. [18]; 0.057–0.068 for Pacific sardine *Sardinops sagax*, 0.055–0.070 for Pacific chub mackerel *Scomber japonicus*, and 0.101 for Northern anchovy *Engraulis mordax* [36]). The degradation rates of freshwater fish were reported as 0.051–0.159 for *L*. *macrochirus* and 0.105 for common carp *Cyprinus carpio* [33,37]. The eDNA degradation may vary among ecosystems (marine or freshwater) and biotic/abiotic conditions [37,38].

In the spatial survey, a significant positive correlation was detected between the estimated distribution of individuals and the eDNA distribution on surface waters, suggesting the possibility that the biomass of this species can be estimated from eDNA concentration. Previously we reported a positive relationship between biomass and eDNA concentration of *T*. *japonicus* using the same eDNA samples as in this study [8]. However, the eDNA concentration of *T*. *japonicus* was affected by eDNA presumably originating from a fish market, and we could therefore detect the positive relation only after removing the effect of the market. On the contrary, *C*. *pacifica* is not targeted in fisheries, and we could therefore detect the direct correlation between the number of individuals and the eDNA concentration in surface water specimens. As such, jellyfish species could serve as favorable model organisms for studying eDNA in marine environments.

The eDNA concentration of *C*. *pacifica* showed significant difference between surface and sea floor water samples, with concentrations reaching as much as ~13 times higher in sea floor water samples than in surface water samples. This difference suggests that more individuals were present at the bottom. Although the vertical distribution of *C*. *pacifica* has not been reported, the moon jellyfish *Aurelia aurita*, which is the main prey of sea nettles [39], typically avoid surface water during daytime and are abundant at a depth of 5–15 m (Yasuda 1971, Suzuki et al. 2016). Therefore, the high eDNA concentration may represent a dense aggregation of the present target species on the sea floor. Further, although the eDNA of jellyfish might precipitate, that of *T*. *japonicus* showed no significant difference between the surface and sea floor samples. The vertical dynamics of eDNA could constitute a future research topic for eDNA studies.

The results of our temporal survey showed the correlation between the numbers of jellyfish observed and the eDNA concentration (Fig 4B). The occurrence of *C*. *pacifica* has been reported as lasting from February to August [40] and from May to August [41] based on intensive plankton net surveys in Tokyo Bay, and our visual observations coincided with these studies. The eDNA concentration of the jellyfish also increased in late spring, peaked in early summer, and decreased in mid-summer. From these results, it appears that the eDNA concentration of *C*. *pacifica* adequately represents the seasonal prevalence of this species (Fig 4B). The recorded eDNA concentrations of six (three from surface and three from bottom) samples correlated with each other, and therefore a single surface sample may reveal changes of jellyfish density in the ocean.

In August, we observed almost no presence of *C*. *pacifica* by visual observation, and no eDNA was detected from surface samples; however, eDNA was detected from sea floor samples. These results suggest that there were jellyfish individuals in the water column although we could not visually observe from the pier. Results of the degradation experiments revealed that 44% of jellyfish eDNA degrade within 24 h. In the temporal survey, the intervals between water samplings were no less than 20 days, suggesting that eDNA concentration was not affected by the remaining eDNA from the previous sampling date. Therefore, the presence of eDNA can be interpreted as representing the presence of the species within a few days of sampling, as shown in previous studies [38]. Actually, eDNA concentration was highly correlated with the summed numbers of individuals observed at the sampling day and the previous one to four days. These results suggest that we can more sensitively detect the presence of a target jellyfish by eDNA analysis than by visual observation from the surface, because jellyfish are present in the water column, which cannot be observed directly.

In this study, we showed that eDNA concentration and jellyfish abundance were correlated both in spatial and temporal surveys, suggesting that estimating abundance or biomass from the eDNA concentration would be possible. *C*. *pacifica* intermittently shows explosive increases in abundance, and an eDNA analysis would provide a forecast of such blooms by estimating abundance or biomass at an early stage of explosion. Additionally, if we can know the distribution of polyps, the explosive increase would be suppressed by removing polyps. This eDNA detection approach would be applicable to other jellyfish species, and it will be especially useful if we can know the distribution of highly venomous species such as box jellyfish represented by *Chironex fleckeri* (also known as sea wasp) and *C*. *yamaguchii* (habu-kurage in Japanese). We hope that the development of eDNA methods for jellyfish will contribute to mitigating the direct and indirect human damages from jellyfish blooms.

## Supporting information

S1 File The raw data for each Figure. The raw qPCR data and the numbers of observed individuals are shown in each sheet.
(XLSX)
